# Reduced Clinical Target Volume Margins in Glioblastoma: Exploratory Evidence Supporting Further Margin Reduction Independent of MGMT Status

**DOI:** 10.3390/brainsci16050458

**Published:** 2026-04-24

**Authors:** Flavio Donnini, Giuseppe Minniti, Salvatore Chibbaro, Giulio Bagnacci, Armando Perrella, Giuseppe Battaglia, Giovanni Rubino, Pierpaolo Pastina, Tommaso Carfagno, Marta Vannini, Maria Antonietta Mazzei, Alfonso Cerase, Paolo Tini

**Affiliations:** 1 Unit of Radiation Oncology, Department of Medicine, Surgery and Neurosciences, University of Siena, 53100 Siena, Italy; flavio.donnini@student.unisi.it (F.D.); giuseppe.battaglia@ao-siena.toscana.it (G.B.); g.rubino@ao-siena.toscana.it (G.R.); pierpaolo.pastina@ao-siena.toscana.it (P.P.); t.carfagno@ao-siena.toscana.it (T.C.);; 2 Radiation Oncology, Policlinico Umberto I, Department of Radiological, Oncological and Pathological Sciences, “Sapienza” University of Rome, 00161 Rome, Italy; giuseppe.minniti@uniroma1.it; 3 Istituto di Ricovero e Cura a Carattere Scientifico Neuromed, 86077 Pozzilli, Italy; 4 Unit of Neurosurgery, Department of Medicine, Surgery and Neurosciences, University of Siena, 53100 Siena, Italy; salvatore.chibbaro@unisi.it; 5 Diagnostic Imaging Unit, Department of Medical Sciences, Azienda Ospedaliero Universitaria Senese, 53100 Siena, Italy; giulio.bagnacci@unisi.it (G.B.); armando.perrella@unisi.it (A.P.);; 6 Diagnostic and Therapeutic Neuroradiology Unit, University Hospital of Siena, 53100 Siena, Italy

**Keywords:** glioblastoma, radiotherapy, clinical target volume, margin reduction, target margins, recurrence patterns, MGMT promoter methylation, precision radiotherapy

## Abstract

**Highlights:**

**What are the main findings?**

- Reduced GTV-to-CTV margins (<1.5 cm; range 1.0–1.4 cm) were not associated with worse survival or altered recurrence patterns in glioblastoma.
- Recurrences remained predominantly in-field, with overlapping distributions between margin groups and no increase in marginal or out-of-field failures, regardless of MGMT status.

**What are the implications of the main findings?**

- These results support the oncological adequacy of current margin strategies and suggest that further margin reduction may be feasible in selected patients.
- No clear MGMT-dependent differences in recurrence patterns were observed in this exploratory cohort, but the study was not powered to detect interaction effects, and these findings should therefore be interpreted cautiously.

**Abstract:**

**Background:** Clinical target volume (CTV) delineation in glioblastoma remains debated, particularly in the era of modern chemoradiation and image-guided radiotherapy. Whether reduced CTV margins can preserve oncological outcomes without increasing marginal or out-of-field failures remains uncertain. We evaluated the association of the gross tumor volume (GTV)-to-CTV margin with survival, patterns of failure, and its interaction with O6-methylguanine-DNA methyltransferase (MGMT) promoter methylation status. **Materials and Methods:** We retrospectively analyzed a single-center cohort of patients with glioblastoma treated with conventionally fractionated chemoradiation (58–60 Gy in 29–33 fractions). Patients were categorized into two predefined margin groups: <1.5 cm and 1.5 cm. The primary endpoint was overall survival (OS); secondary endpoints included progression-free survival (PFS) and patterns of failure. Survival was assessed using Kaplan–Meier estimates and Cox regression, including an interaction term with MGMT status. **Results:** Among 102 eligible patients, 95 were included in the margin-based OS analysis. Reduced margins (<1.5 cm; applied range 1.0–1.4 cm) were not associated with worse OS, either overall or within MGMT subgroups. No significant differences were observed in PFS or recurrence patterns, with overlapping distributions and no increase in marginal or out-of-field recurrences. MGMT methylation and gross total resection were independently associated with improved survival, while no statistically significant interaction between margin and MGMT status was detected. **Conclusions:** In this retrospective exploratory cohort, reduced GTV-to-CTV margins were not associated with a clear signal of worse survival or less favorable recurrence patterns. These findings are consistent with the oncological adequacy of margins around 15 mm and justify cautious prospective evaluation of whether further reduction can be achieved safely, including formal assessment of toxicity, neurocognitive outcomes, and quality of life.

## 1. Introduction

Glioblastoma is the most aggressive primary malignant brain tumour in adults and remains associated with a poor prognosis despite multimodal treatment [1]. The current standard of care for newly diagnosed disease consists of maximal safe resection followed by conventionally fractionated radiotherapy with concomitant and adjuvant temozolomide [1]. Although this regimen has remained the therapeutic backbone for nearly two decades, disease progression is common and most patients ultimately relapse [1,2]. MGMT promoter methylation has emerged as one of the most clinically relevant biomarkers in glioblastoma because of both its favourable prognostic value and its association with greater benefit from temozolomide-based therapy [3].

Target volume delineation remains one of the major unresolved issues in glioblastoma radiotherapy. Historically, larger clinical target volume (CTV) margins were intended to account for the infiltrative nature of glioblastoma and for microscopic tumor extension beyond the contrast-enhancing lesion [2]. However, larger CTV expansions inevitably increase the volume of irradiated normal brain and may raise the risk of treatment-related toxicity, including neurocognitive impairment [2,4]. Reflecting the need to balance oncological coverage with normal-tissue sparing, the recent ESTRO-EANO guideline supports a contemporary single-CTV approach based on postoperative contrast-enhanced T1 abnormalities with an isotropic margin of approximately 15 mm, without routine cone-down, together with limited planning target volume margins when image guidance is available [2].

Several retrospective series evaluating limited-margin strategies have reported that most glioblastoma recurrences remain central or in-field, whereas marginal and distant failures are comparatively uncommon [4,5,6,7]. In this context, reduced-margin approaches have not been associated with an increased risk of marginal or distant failure when modern radiotherapy techniques are employed [4,5,6,7]. These findings suggest that current target margins may already be sufficient to encompass clinically relevant microscopic disease extension, raising the question of whether further margin reduction could be feasible without compromising oncological outcomes.

Despite these observations, uncertainty persists regarding the clinical implications of reduced-margin irradiation in routine practice. In particular, it remains unclear whether margin adequacy is influenced by tumor biology [8], particularly MGMT promoter methylation status [9], or whether it is primarily determined by spatial patterns of tumor recurrence. Few real-world studies have jointly assessed survival outcomes, patterns of failure, and the potential modifying role of MGMT in the context of margin reduction strategies [4,8]. Addressing this question is clinically relevant, as a reduced-margin approach is meaningful only if it does not compromise survival or shift recurrence patterns outside the high-dose region.

Building on prior retrospective series, including our previous institutional report primarily focused on feasibility of CTV reduction based on patterns-of-failure analysis, the present study was designed to extend that literature by jointly evaluating overall survival, progression-free survival, spatial recurrence patterns, and exploratory interaction with MGMT promoter methylation status in a contemporary real-world cohort. We therefore retrospectively analyzed a single-center cohort of patients with glioblastoma treated with chemoradiation to assess whether reduced GTV-to-CTV margins were associated with survival outcomes or recurrence patterns and whether any signal of differential effect emerged across MGMT-defined subgroups.

## 2. Materials and Methods

### 2.1. Study Design and Ethics

We conducted a retrospective single-center observational study based on an institutional database of patients with glioblastoma treated with radiotherapy at our institution. The study was designed to assess the association of the GTV-to-CTV margin with survival outcomes and patterns of failure in patients undergoing chemoradiation. Particular emphasis was placed on evaluating the consistency between survival endpoints and spatial recurrence patterns. All data were retrospectively retrieved from routinely maintained institutional clinical, radiological, and treatment records. The study was conducted in accordance with the Declaration of Helsinki and was approved by the local Ethics Committee of Azienda Ospedaliera Universitaria Senese, Area Vasta Sud-Est (protocol code: GLIOMARKERSOBS_2025-1; approval date: 6 May 2025).

### 2.2. Patient Selection

Patients were eligible if they had a diagnosis of glioblastoma and were treated with radiotherapy at our institution. For the primary analysis, we predefined a cohort of patients treated with conventionally fractionated chemoradiation to a total dose of 58–60 Gy delivered in 29–33 fractions between 1 January 2022 and 31 August 2025. To be included in the primary cohort, patients were required to have available data on radiotherapy start date, total prescribed dose, number of fractions, GTV-to-CTV margin, and overall survival. Patients treated with clearly palliative or markedly hypofractionated regimens outside the predefined dose/fractionation range were excluded from the primary cohort. For the primary exposure analysis, patients were categorized into two prespecified GTV-to-CTV margin groups reflecting the two most clinically distinct approaches represented in our institutional practice: <1.5 cm and 1.5 cm. This approach was intended to enable a clearer assessment of whether distinct margin strategies were associated with differences in survival or spatial recurrence behavior. For multivariable analyses, a complete-case approach was used for the selected covariates. For progression-free survival and pattern-of-failure analyses, only patients with available and classifiable data for the respective endpoints were included.

### 2.3. Treatment and Target Delineation

All patients underwent multidisciplinary evaluation before treatment. Surgical management consisted of biopsy, subtotal resection, or gross total resection, according to neurosurgical judgment and postoperative imaging assessment. After surgery, patients received conventionally fractionated external beam radiotherapy with concurrent temozolomide according to institutional practice and contemporary standard management of glioblastoma [1].

Radiotherapy was delivered using external beam image-guided techniques (VMAT with daily IGRT) according to institutional protocols. Treatment planning was based on postoperative planning CT, co-registered with postoperative brain MRI for target delineation. The gross tumor volume (GTV) was defined based on postoperative T1 contrast-enhancing residual tumor and/or the resection cavity. The clinical target volume (CTV) was generated by isotropic expansion of the GTV, with anatomical editing when required to respect natural barriers to tumor spread.

According to contemporary ESTRO–EANO recommendations, a CTV margin of approximately 15 mm is considered a standard approach [2]. However, in routine clinical practice at our institution, margin selection was individualized and, in selected cases, reduced margins (<1.5 cm) were adopted. Within the reduced-margin group, applied margins ranged from 1.0 to 1.4 cm, and no margins below 1 cm were used. These decisions were not protocol-mandated but reflected clinician judgment in selected cases, particularly when anatomical boundaries, image co-registration quality, and the goal of limiting unnecessary irradiation of uninvolved brain tissue, especially in eloquent or functionally critical regions, supported a more conservative expansion. Because this process was retrospective and non-randomized, not all determinants of margin choice could be fully reconstructed from the available dataset. The resulting margin distribution therefore reflects real-world decision-making rather than a protocol-driven contouring strategy. A planning target volume (PTV) margin of 3 mm was then added according to immobilization and image-guidance practice. Patients were treated with conventionally fractionated schedules of 58–60 Gy in 29–33 fractions, with concurrent temozolomide administered according to standard practice [1,3].

The key exposure variable of the present study was the recorded GTV-to-CTV margin, expressed in centimeters. For the primary comparison, this variable was prespecified as a binary exposure (<1.5 cm vs. 1.5 cm), enabling the evaluation of clinically meaningful differences between reduced and guideline-based margin strategies. This binary specification was chosen to reflect the two most clinically distinct margin strategies represented in institutional practice. Because applied margins within the reduced-margin group were narrowly distributed (1.0–1.4 cm), the study was not designed to support finer stratification or continuous modeling of the margin variable.

### 2.4. Molecular and Clinical Variables

Clinical and molecular variables were retrieved from institutional medical, radiological, surgical, and radiotherapy records. Baseline variables included age at diagnosis, sex, Karnofsky Performance Status (KPS), extent of resection, focality, and selected treatment-related variables. Extent of resection was classified based on neurosurgical documentation and postoperative contrast-enhanced MRI assessment. Biopsy was defined as a diagnostic procedure without meaningful tumor debulking. Gross total resection was defined as the absence of residual contrast-enhancing tumor on postoperative imaging, whereas subtotal resection indicated residual contrast-enhancing tumor after surgical debulking. MGMT promoter methylation status was recorded as methylated or unmethylated according to institutional pathology reports and was included both as an independent biomarker and as a potential effect modifier in interaction analyses. The interaction analysis was specifically intended to explore whether the effect of margin strategy differed according to tumour molecular profile. Covariates included in multivariable analyses were selected a priori based on clinical relevance and data availability.

### 2.5. Endpoints

The primary endpoint was overall survival (OS), defined as the time from histological diagnosis to death from any cause. Patients alive at the time of last follow-up were censored at the date of last clinical contact.

Secondary endpoints were progression-free survival (PFS) and pattern of failure. PFS was defined as the time from histological diagnosis to radiological progression or death, whichever occurred first. Progression status was retrospectively determined from institutional follow-up imaging records and clinical documentation generated during routine neuro-oncological care, with reference to contemporary Response Assessment in Neuro-Oncology (RANO) principles when applicable [10,11]. Patients without sufficiently reliable information to determine progression status were excluded from PFS-specific analyses.

Follow-up consisted of regular clinical and neuroradiological assessments according to institutional practice. Brain MRI was typically performed approximately 4–6 weeks after radiotherapy and then at regular intervals thereafter, unless earlier imaging was clinically indicated. The joint evaluation of survival outcomes and spatial recurrence patterns was considered essential to assess the oncological adequacy of different margin strategies.

### 2.6. Pattern of Failure Assessment

Pattern-of-failure analysis was performed in the subset of patients with available and classifiable data on first recurrence. Recurrences were retrospectively categorized according to their spatial relationship with the high-dose irradiated volume, using a classification framework commonly adopted in glioblastoma recurrence studies and consistent with the previously published institutional series [4,8,9]. Whenever sufficient imaging and treatment data were available, follow-up MRI scans were co-registered with the original radiotherapy treatment plan using RayStation^®^ software version 2025 (RaySearch Laboratories AB, Stockholm, Sweden). Spatial classification referred to the first documented recurrence. Recurrence assessment was primarily performed by a radiation oncologist, while doubtful or uncertain cases were additionally reviewed with a neuroradiologist. Formal blinding to margin group was not implemented, and interobserver variability was not formally assessed. Specifically, recurrences were classified as in-field when at least 80% of the recurrent tumour volume was located within the 95% isodose, as marginal when 20–80% of the recurrent tumour volume was located within the 95% isodose, and as out-of-field when less than 20% of the recurrent tumour volume was located within the 95% isodose. Cases lacking sufficient radiological or treatment information for reliable spatial classification were excluded from this endpoint analysis. For exploratory purposes, recurrence patterns were also dichotomized into in-field versus non-in-field recurrences, with the latter category including both marginal and out-of-field failures. This approach was intended to provide a clinically intuitive assessment of whether reduced margins were associated with a shift in recurrence outside the high-dose region.

### 2.7. Statistical Analysis

Baseline characteristics were summarized descriptively and compared between the two prespecified GTV-to-CTV margin groups (<1.5 cm vs. 1.5 cm). Continuous variables were reported as mean and standard deviation or median and interquartile range, as appropriate, and were compared using Student’s *t*-test or the Mann–Whitney U test according to distributional assumptions. Categorical variables were summarized as counts and percentages and were compared using the chi-square test or Fisher’s exact test, as appropriate.

Overall survival (OS) and progression-free survival (PFS) were estimated using the Kaplan–Meier method and compared between groups using the log-rank test. Survival analyses were first performed in the overall cohort and then stratified according to MGMT promoter methylation status.

Multivariable analysis for OS was performed using a Cox proportional hazards regression model. The model included GTV-to-CTV margin group, MGMT promoter methylation status, their interaction term, age, baseline Karnofsky Performance Status (KPS), and extent of resection. These covariates were selected a priori based on established clinical relevance and data availability. To limit model complexity relative to the number of observed events, multivariable adjustment was restricted to a small set of clinically established variables selected a priori rather than through data-driven variable selection procedures. The interaction between margin group and MGMT status was analyzed to explore potential effect modification; however, this analysis was considered exploratory and hypothesis-generating. Because concurrent temozolomide was administered in nearly all evaluable patients, this variable showed insufficient variability to provide meaningful information in the final multivariable model and was therefore not retained. Hazard ratios (HRs) with 95% confidence intervals (CIs) were reported. A complete-case approach was used for multivariable analysis. Proportional hazards assumptions were assessed using Schoenfeld residuals.

For recurrence-pattern analyses, Fisher’s exact test was used to compare the distribution of the three recurrence categories between margin groups, given the limited number of marginal and out-of-field events. Fisher’s exact test was also used for the binary comparison between in-field and non-in-field recurrences.

Given the observational design, emphasis was placed on the consistency of findings across multiple endpoints rather than on any single statistical comparison.

All tests were two-sided, and a *p*-value < 0.05 was considered statistically significant. Statistical analyses were performed in R version 4.5.2 (R Foundation for Statistical Computing, Vienna, Austria). Survival analyses and graphical outputs were generated using standard R packages.

## 3. Results

### 3.1. Patient Selection and Baseline Characteristics

**Figure 1:**
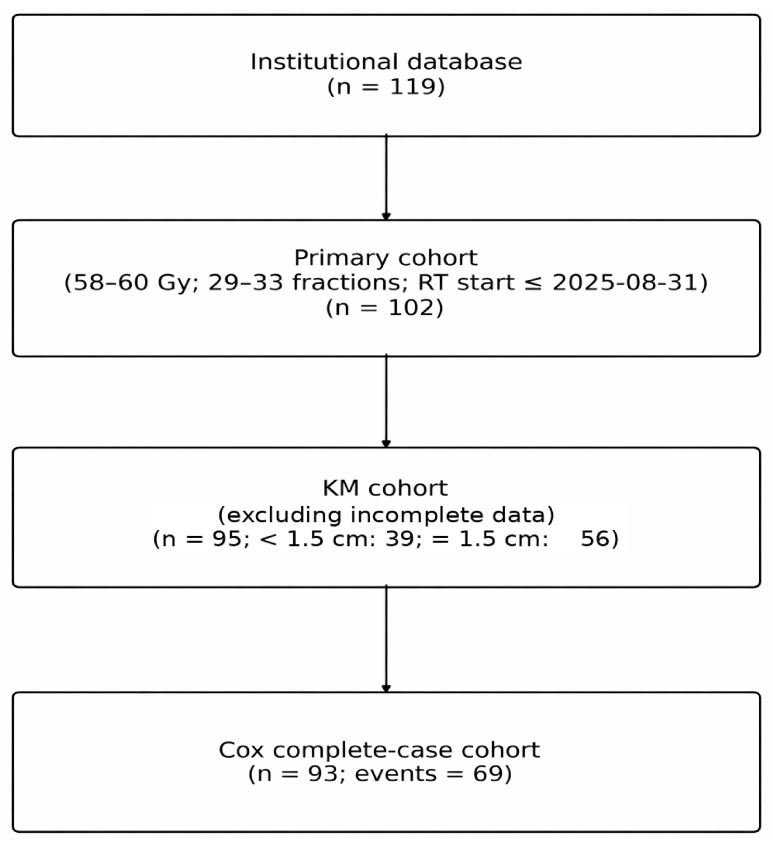
Flowchart of patient selection and derivation of the analytic cohorts. Of 119 patients identified in the institutional database, 102 met the predefined eligibility criteria for the primary cohort. Ninety-five patients in the two prespecified margin groups were included in the margin-based OS analysis, 93 in the complete-case Cox model, 91 in the PFS cohort, and 68 in the pattern-of-failure cohort, depending on the availability of covariate, progression, and classifiable spatial recurrence data.

A total of 119 patients were identified in the institutional database. Of these, 102 met the predefined criteria for the primary cohort. The margin-based OS cohort further excluded patients outside the two prespecified margin groups, whereas the complete-case Cox, PFS, and pattern-of-failure cohorts additionally depended on the availability of covariate, progression, and classifiable recurrence-pattern data, respectively. For the complete-case multivariable Cox analysis, 93 patients were evaluable, with 69 death events observed. The derivation of the analytic cohorts is summarized in Figure 1.

After exclusion of patients outside the two prespecified margin groups, 95 patients were included in the primary margin-based OS analysis, including 39 treated with a GTV-to-CTV margin of <1.5 cm and 56 treated with a margin of 1.5 cm (Baseline characteristics of the complete-case multivariable cohort are summarized in Table 1).

No statistically significant between-group differences were observed in the variables reported. Mean age was 59.2 years in the <1.5 cm group and 62.1 years in the 1.5 cm group. The proportions of MGMT-methylated tumours were similar across groups, as was the distribution of biopsy, subtotal resection, and gross total resection.

### 3.2. Overall Survival and Progression-Free Survival According to CTV Margin

**Figure 2:**
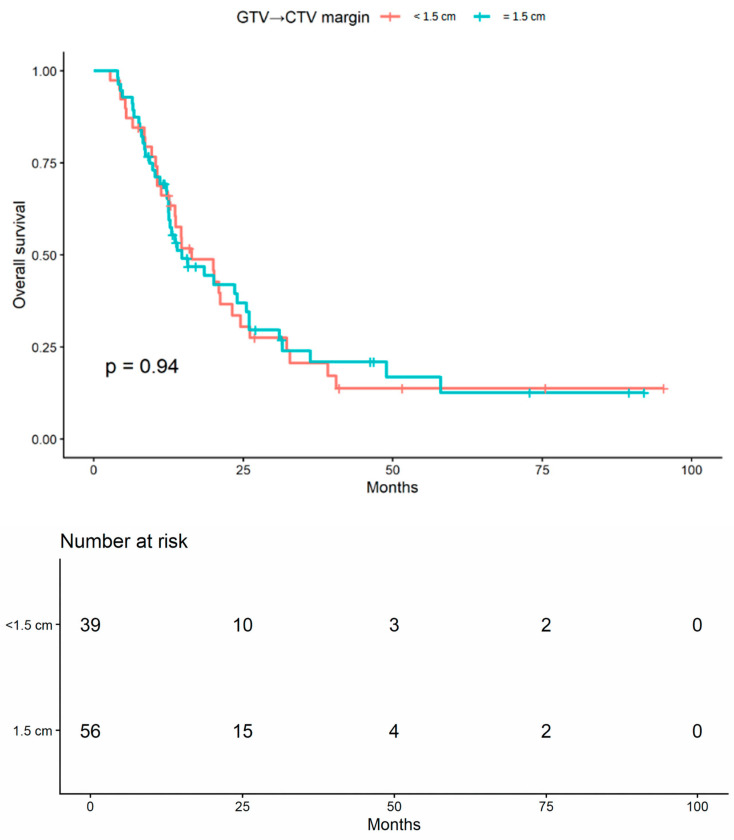
Kaplan–Meier curves for overall survival according to GTV-to-CTV margin group (<1.5 cm vs. 1.5 cm) in the primary margin-based OS cohort. The *p*-value was calculated using the log-rank test.

In unadjusted survival analyses, no statistically significant difference in overall survival (OS) was observed between patients treated with a GTV-to-CTV margin of <1.5 cm and those treated with a margin of 1.5 cm (log-rank *p* = 0.944; Figure 2).

**Figure 3:**
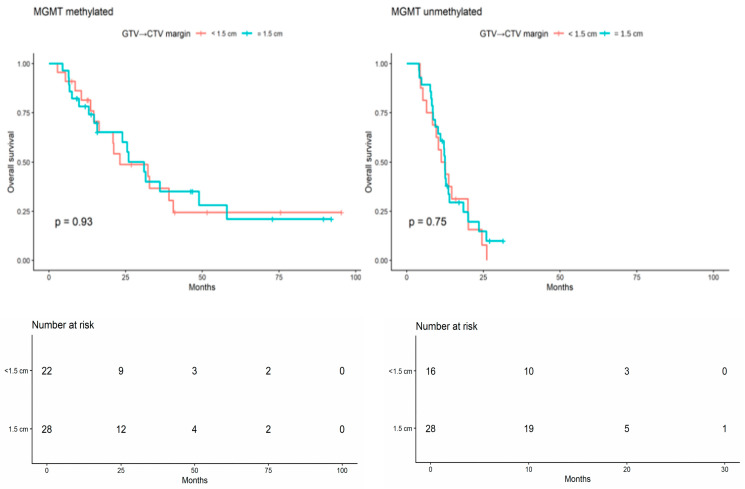
Kaplan–Meier curves for overall survival according to GTV-to-CTV margin group (<1.5 cm vs. 1.5 cm), shown separately within MGMT-methylated and MGMT-unmethylated subgroups.

When the analysis was stratified by MGMT promoter methylation status, no statistically significant difference in OS was observed within either subgroup (MGMT-methylated: log-rank *p* = 0.926; MGMT-unmethylated: log-rank *p* = 0.752; Figure 3). Overall, these unadjusted findings did not suggest inferior OS among patients treated with the reduced-margin approach, either in the full cohort or within MGMT-defined subgroups.

**Figure 4:**
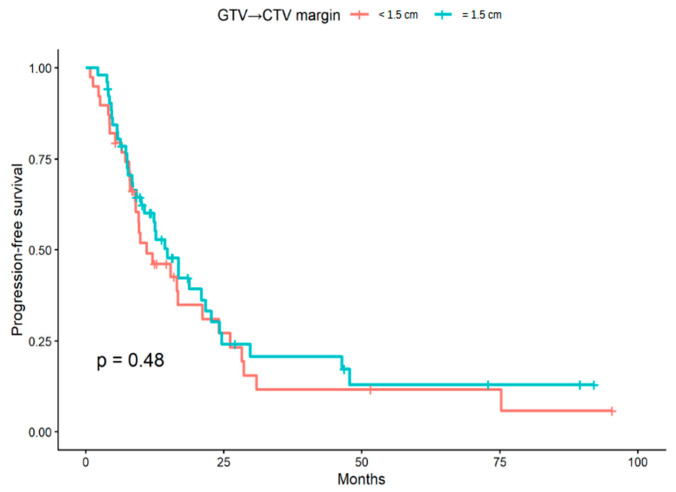

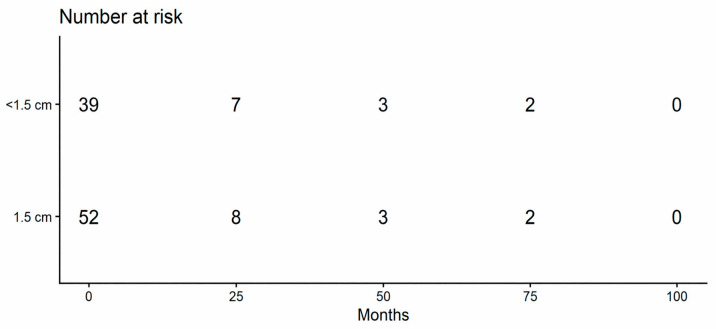
Kaplan–Meier curves for progression-free survival according to GTV-to-CTV margin group (<1.5 cm vs. 1.5 cm). PFS was calculated from the date of histological diagnosis. The *p*-value was obtained using the log-rank test.

Progression-free survival (PFS) was evaluable in 91 patients after exclusion of cases with uncertain progression status. Of these, 39 patients belonged to the GTV-to-CTV margin < 1.5 cm group and 52 to the 1.5 cm group. In unadjusted Kaplan–Meier analysis, no statistically significant difference in PFS was observed between the two margin groups (log-rank *p* = 0.48; Figure 4).

**Figure 5:**
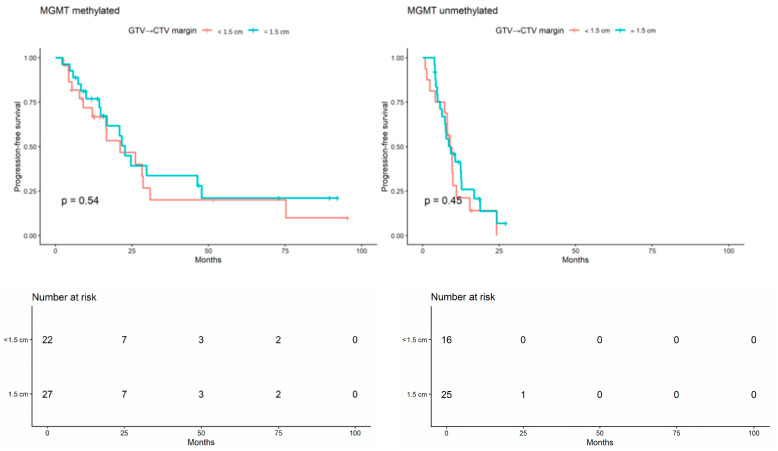
Kaplan–Meier curves for progression-free survival according to GTV-to-CTV margin group (<1.5 cm vs. 1.5 cm), shown separately within MGMT-methylated and MGMT-unmethylated subgroups.

When the analysis was stratified by MGMT promoter methylation status, no statistically significant difference in PFS was observed in either subgroup (MGMT-methylated: log-rank *p* = 0.54; MGMT-unmethylated: log-rank *p* = 0.45; Figure 5). Overall, these unadjusted findings did not suggest inferior PFS among patients treated with the reduced-margin approach, either in the full cohort or within MGMT-defined subgroups.

### 3.3. Multivariable Analysis

**Figure 6:**
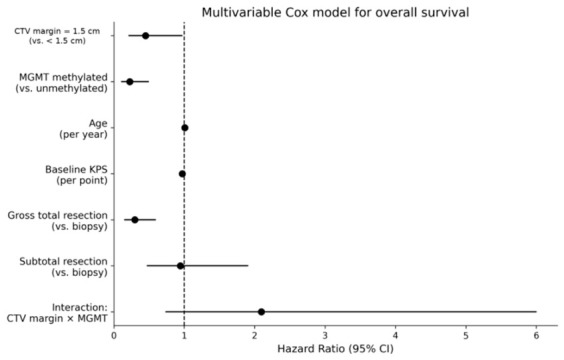
Forest plot of the multivariable Cox proportional hazards model for overall survival. Forest plot showing hazard ratios and 95% confidence intervals for the covariates included in the complete-case multivariable Cox model. The model included GTV-to-CTV margin group, MGMT promoter methylation status, the interaction between margin group and MGMT status, age, baseline Karnofsky Performance Status, and extent of resection.

A multivariable Cox proportional hazards model was used to explore whether the association between margin strategy and overall survival (OS) was influenced by MGMT status and major clinical covariates. In this analysis, MGMT promoter methylation was independently associated with improved overall survival (HR 0.23, 95% CI 0.10–0.50; *p* < 0.001), and gross total resection was also associated with improved survival compared with biopsy (HR 0.30, 95% CI 0.15–0.60; *p* < 0.001). Baseline KPS was not statistically significant, although higher values were associated with a lower estimated hazard (HR 0.97 per point increase, 95% CI 0.94–1.00; *p* = 0.051), whereas age was not significantly associated with outcome. Within the parameterization of the interaction model, the main effect for CTV margin represents the comparison within the MGMT-unmethylated reference group. Under this parameterization, the model estimated lower hazard for patients treated with a 1.5 cm margin than for those treated with a margin < 1.5 cm (HR 0.45, 95% CI 0.21–0.97; *p* = 0.043). In contrast, the derived hazard ratio for the same margin comparison in MGMT-methylated tumours was 0.94 (95% CI 0.45–1.99; *p* = 0.879). However, the interaction between margin group and MGMT promoter methylation was not statistically significant (*p* = 0.168). These subgroup-specific estimates should therefore be considered exploratory and model-dependent and should not be interpreted as evidence of a true differential margin effect according to MGMT status. The proportional hazards assumption was not violated, as assessed by Schoenfeld residuals (global test *p* = 0.284). No significant departures from proportionality were detected for margin group (*p* = 0.311) or MGMT status (*p* = 0.452). The results of the multivariable Cox model are summarized in Table 2 and graphically displayed in Figure 6.

### 3.4. Pattern of Failure Analysis

**Figure 7:**
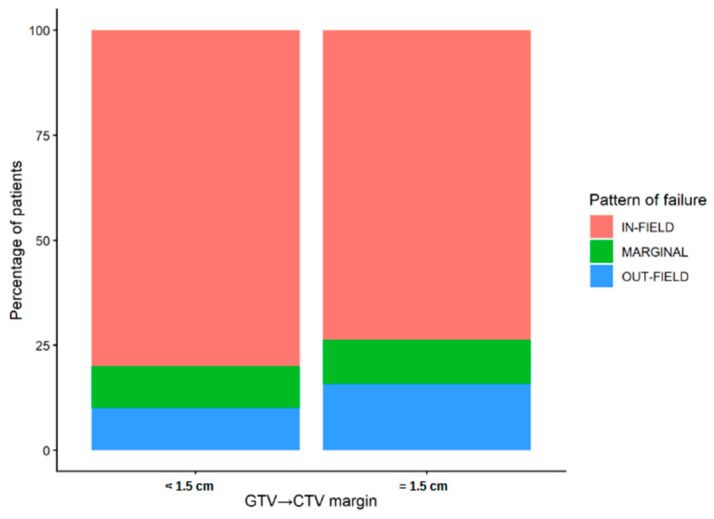
Distribution of recurrence patterns according to GTV-to-CTV margin group. Stacked bar plot showing the percentage distribution of in-field, marginal, and out-of-field recurrences among patients treated with a GTV-to-CTV margin < 1.5 cm versus 1.5 cm (Fisher’s exact test *p* = 0.913).

Pattern-of-failure analysis was available in 68 patients, including 30 in the GTV-to-CTV margin < 1.5 cm group and 38 in the 1.5 cm group. Overall, 52 recurrences were classified as in-field, 7 as marginal, and 9 as out-of-field. The distribution of recurrence patterns did not differ significantly between margin groups (Fisher’s exact test *p* = 0.913; Figure 7). When recurrence patterns were dichotomized as in-field versus non-in-field, no statistically significant between-group difference was observed (24 vs. 6 in the <1.5 cm group and 28 vs. 10 in the 1.5 cm group, respectively; Fisher’s exact test *p* = 0.579). Overall, these analyses did not provide evidence of an increased frequency of recurrences outside the expected high-dose region in the reduced-margin group.

**Figure 8:**
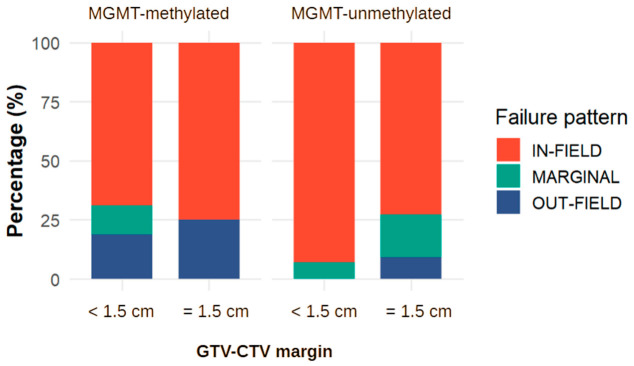
Distribution of recurrence patterns (in-field, marginal, and out-of-field) according to GTV-to-CTV margin (<1.5 cm vs. 1.5 cm), stratified by MGMT promoter methylation status. Percentages are shown within each subgroup. Across both MGMT-methylated and unmethylated cohorts, recurrence patterns were predominantly in-field and showed overlapping distributions between margin groups, with no apparent increase in marginal or out-of-field failures in the reduced-margin group.

Exploratory analyses stratified by MGMT promoter methylation status were performed in the subset of patients with available molecular data and classifiable recurrence patterns (n = 68; 32 MGMT-methylated and 36 MGMT-unmethylated). Within the MGMT-methylated subgroup, the distribution of recurrence patterns was similar between margin groups (<1.5 cm: 11 in-field, 2 marginal, 3 out-of-field; 1.5 cm: 12 in-field, 0 marginal, 4 out-of-field; Fisher’s exact test *p* = 0.525). Likewise, in the MGMT-unmethylated subgroup, no statistically significant difference in recurrence distribution was observed (<1.5 cm: 13 in-field, 1 marginal, 0 out-of-field; 1.5 cm: 16 in-field, 4 marginal, 2 out-of-field; *p* = 0.348) (Figure 8).

Consistent findings were obtained in the binary analysis (in-field vs. non-in-field). No significant differences were detected between margin groups in either the MGMT-methylated (*p* = 1.000) or MGMT-unmethylated subgroup (*p* = 0.209), although estimates were imprecise due to the limited number of non-in-field events.

Overall, these exploratory subgroup analyses did not identify any signal suggesting an increased risk of marginal or out-of-field recurrence associated with reduced margins, regardless of MGMT promoter methylation status. Across both molecular subgroups, most recurrences remained in-field, with largely overlapping distributions between margin strategies.

## 4. Discussion

The present study evaluated the association between GTV-to-CTV margin strategy and clinical outcomes in a retrospective single-centre cohort of patients with glioblastoma treated with chemoradiation. Reduced GTV-to-CTV margins (<1.5 cm) were not associated with worse overall survival or progression-free survival, nor with an increased proportion of marginal or out-of-field recurrences. These findings were broadly consistent across MGMT-defined subgroups, although the interaction analysis was exploratory and not powered to detect effect modification reliably.

Within the reduced-margin group, applied margins ranged from 1.0 to 1.4 cm. Although the present study was not designed to define a lower safe threshold, the absence of an obvious adverse signal across this range suggests that reduced-margin strategies warrant further prospective study.

Most recurrences in both groups remained within the high-dose region, supporting the view that glioblastoma failure is driven predominantly by aggressive local disease biology rather than by inadequate geometric coverage of subclinical extension alone.

These findings are consistent with prior reports demonstrating that limited-margin strategies do not increase marginal or distant failures when modern radiotherapy techniques are used [4,5,6,7,11]. McDonald et al. and Paulsson et al. reported no excess of marginal recurrences with reduced margins, while Gebhardt et al. similarly observed a low rate of out-of-field failures [5,6,7]. More recently, Minniti et al. suggested that margin reduction may preserve recurrence patterns while reducing unnecessary irradiation of normal brain tissue [4]. In this context, the main added contribution of the present study lies in the integrated evaluation of survival outcomes, recurrence patterns, and exploratory MGMT-related effect modification within a contemporary real-world cohort.

Conceptually, the present findings suggest that target-margin adequacy in glioblastoma radiotherapy may be more closely related to spatial recurrence behaviour than to MGMT-defined tumour biology. However, because the interaction analysis was exploratory and underpowered, this interpretation should remain cautious and should not be taken as definitive evidence that margin effects are independent of molecular context.

Clinically, these findings are consistent with the adequacy of margins around 15 mm, as recommended by contemporary guidelines [2]. At the same time, the absence of a clear signal of increased marginal or distant failure in the reduced-margin group supports cautious prospective evaluation of whether further reduction may be achievable in selected settings. Any meaningful clinical advantage of this approach, however, will ultimately need to be demonstrated through formal assessment of neurocognitive function, treatment-related toxicity, and patient-reported quality of life, rather than through tumour-control endpoints alone.

An additional point of context concerns the evolving surgical literature on supratotal resection in glioblastoma. Increasing evidence from specialized centers suggests that, in selected cases and when functionally feasible, resection beyond the contrast-enhancing tumor boundaries may improve oncological outcomes [12,13]. At the same time, this field remains characterized by heterogeneous definitions and predominantly retrospective evidence [12]. No patients in the present cohort underwent supratotal resection, and therefore this surgical strategy could not be assessed in our analysis. At first glance, the coexistence of increasing surgical aggressiveness and reduced irradiation volumes may appear paradoxical. However, these approaches may instead be viewed as complementary expressions of treatment personalization: surgery seeks to maximize safe local cytoreduction, whereas modern radiotherapy seeks to minimize unnecessary exposure of uninvolved brain while maintaining oncological adequacy in regions at highest risk of recurrence.

The multivariable analysis requires cautious interpretation. Because the model included an interaction term, the main effect for margin reflects the comparison within the MGMT-unmethylated reference group, and subgroup-specific estimates depend on model parameterization. Although the point estimate in that subgroup appeared to favour 1.5 cm margins, the interaction term was not statistically significant, and no consistent signal of harm associated with reduced margins emerged across OS, PFS, and recurrence-pattern analyses. Accordingly, this apparent subgroup signal is more plausibly interpreted as exploratory and potentially attributable to chance, residual confounding by indication, or model instability in a relatively small dataset than as evidence of a true biologically meaningful differential effect.

Overall, the main strength of the present study lies in the concordance of findings across survival and spatial recurrence endpoints within a contemporary real-world cohort. These results should be viewed as supportive but not definitive and are best interpreted as a rationale for prospective testing rather than as a basis for immediate modification of standard contouring practice.

## 5. Limitations

This study has several limitations. First, its retrospective single-centre design makes the analysis inherently susceptible to selection bias and residual confounding. Margin assignment was not randomized and may have been influenced by physician preference, treatment era, anatomical considerations, imaging features, or perceived eloquence, not all of which could be fully reconstructed from the available dataset. Although baseline characteristics were broadly comparable between groups, confounding by indication cannot be excluded.

Second, the overall sample remained modest for subgroup, interaction, and recurrence-pattern analyses. Although the complete-case overall survival analysis included 69 death events, the study may still have been underpowered to detect small-to-moderate survival differences between margin strategies, uncommon shifts toward marginal or out-of-field recurrence, or true interaction effects according to MGMT status. Accordingly, the absence of statistically significant differences should not be interpreted as proof that such differences do not exist. In addition, because reduced margins ranged only from 1.0 to 1.4 cm, the study was designed as a comparison between two clinically distinct strategy groups rather than as an analysis intended to define an optimal lower threshold or support finer subgrouping within the reduced-margin range.

Third, the multivariable analysis was based on a complete-case approach and should be regarded as exploratory, given the modest ratio between observed events and estimated parameters. Moreover, progression-free survival and pattern-of-failure assessments relied on retrospective review of routine clinical and imaging data, without centralized adjudication or formal blinded review. In glioblastoma, this is particularly relevant because retrospective RANO-based progression assessment may be affected by treatment-related imaging changes and pseudoprogression. Pattern-of-failure analysis was also available only in a subset of patients, and interobserver variability was not formally quantified.

Finally, the study did not include neurocognitive outcomes, treatment-related toxicity, or patient-reported quality of life, which are central to the clinical rationale for margin reduction. In the absence of a clear survival difference, the value of smaller margins would ideally be demonstrated through reduced morbidity or improved patient-reported outcomes. In addition, no patients in this cohort underwent supratotal resection, and the present findings therefore cannot be extrapolated to treatment paradigms incorporating more extensive surgical approaches.

Despite these limitations, the consistency observed across survival and spatial recurrence endpoints supports the biological plausibility of the overall findings.

## 6. Conclusions

In this retrospective exploratory single-centre cohort of patients with glioblastoma treated with chemoradiation, reduced GTV-to-CTV margins (<1.5 cm; applied range 1.0–1.4 cm) were not associated with a clear signal of worse survival or less favourable recurrence patterns. These findings support cautious prospective evaluation of reduced-margin strategies but should be considered hypothesis-generating rather than definitive. Future studies should incorporate neurocognitive outcomes, treatment-related toxicity, and patient-reported quality of life to determine whether margin reduction provides a meaningful clinical benefit beyond preserved tumour control.

## Figures and Tables

**Table 1:** Baseline characteristics of the complete-case–cohort. *p*-values refer to between-group comparisons.

Variable		CTV Margin < 1.5 cm (n = 37)	CTV Margin 1.5 cm (n = 56)	*p*-Value
Age, years (mean ± SD)		59.24 (10.61)	62.13 (9.91)	0.184
Baseline KPS (mean ± SD)		88.65 (9.76)	90.00 (8.94)	0.493
MGMT promoter methylation status, n (%)	Unmethylated	16 (43.2)	28 (50.0)	0.670
	Methylated	21 (56.8)	28 (50.0)	
Extent of resection, n (%)	Biopsy	7 (18.9)	8 (14.3)	0.612
	Gross total resection	19 (51.4)	26 (46.4)	
	Subtotal resection	11 (29.7)	22 (39.3)	

**Table 2:** Multivariable Cox proportional hazards model for overall survival in the complete-case–cohort. The main effect for CTV margin represents the margin comparison within the MGMT-unmethylated reference group because the model included an interaction term between CTV margin group and MGMT promoter methylation status. Hazard ratios are reported with 95% confidence intervals.

Covariate	Hazard Ratio (95% CI)	*p*-Value
CTV margin 1.5 cm (vs. <1.5 cm), within the MGMT-unmethylated reference group	0.45 (0.208–0.975)	0.0429
MGMT methylated (vs. unmethylated)	0.226 (0.103–0.498)	0.000225
Age, years	1.007 (0.981–1.034)	0.583
Baseline KPS, per point	0.972 (0.944–1)	0.0514
Gross total resection (vs. biopsy)	0.299 (0.149–0.598)	0.000641
Subtotal resection (vs. biopsy)	0.946 (0.469–1.911)	0.878
Interaction: CTV margin × MGMT methylation	2.096 (0.732–6.005)	0.168
Derived effect: CTV margin 1.5 cm in MGMT-methylated subgroup	0.944 (0.449–1.985)	0.879

## Data Availability

The data presented in this study are not publicly available due to privacy and institutional restrictions. De-identified data may be available from the corresponding author on reasonable request and subject to approval by the local Ethics Committee and institutional policies.
